# Crystal structure of silver strontium copper orthophosphate, AgSr_4_Cu_4.5_(PO_4_)_6_

**DOI:** 10.1107/S2056989020000109

**Published:** 2020-01-10

**Authors:** Jamal Khmiyas, Elhassan Benhsina, Said Ouaatta, Abderrazzak Assani, Mohamed Saadi, Lahcen El Ammari

**Affiliations:** a Laboratoire de Chimie Appliquée des Matériaux, Centre des Sciences des Matériaux, Faculty of Sciences, Mohammed V University in Rabat, Avenue Ibn Batouta, BP 1014, Rabat, Morocco

**Keywords:** crystal structure, AgSr_4_Cu_4.5_(PO_4_)_6_, transition metal phosphate, hydro­thermal synthesis, layered structure

## Abstract

Crystals of the new compound, AgSr_4_Cu_4.5_(PO_4_)_6_, were grown successfully by the hydro­thermal process. The asymmetric unit of the crystal structure of the title compound contains 40 independent atoms (4 Sr, 4.5 Cu, 1 Ag, 6 P and 24 O), which are all in general positions except for one Cu atom, which is located on an inversion centre. The Cu atoms are arranged in CuO_*n*_ (*n* = 4 or 5) polyhedra, linked through common oxygen corners to build a rigid three-dimensional motif. The connection of these copper units is assured by PO_4_ tetra­hedra. This arrangement allows the construction of layers extending parallel to the (100) plane and hosts suitable cavities in which Ag^+^ and Sr^2+^ cations are located. The crystal-structure cohesion is ensured by ionic bonds between the silver and strontium cations and the oxygen anions belonging to two adjacent sheets. Charge-distribution analysis and bond-valence-sum calculations were used to validate the structural model.

## Abstract 2

The transition-metal orthophosphate, AgSr_4_Cu_4.5_(PO_4_)_6_, crystallizes in an original structure characterized by two trimers [Cu_3_O_12_] linked by PO_4_ tetra­hedra to form layers. The strontium and silver cations located between the layers ensure the cohesion of the crystal structure.

## Chemical context   

The growing role of metal orthophosphates based on PO_4_ and *M*O_*n*_ (where *M* is a metal cation) structural units is closely related to their ability to adopt different spatial arrangements. As has been pointed out previously, their physical and chemical properties, dynamic flexibility attributes and structural behaviour (Hadrich *et al.*, 2001 ▸) can be correlated with the ionic radius of the metal cation (Jeżowska-Trzebiatowska *et al.*, 1980 ▸). Furthermore, the ability of these metal cations to adopt different oxidation states as well as various coordination environments leads, in general, to open anionic three-dimensional frameworks. The structures of these classes of materials can easily accommodate a great variety of substituents, anionic and/or cationic, which can have a significant effect on the stability and on the morphology of structures and crystals, as is shown particularly in the apatite family (LeGeros & Tung, 1983 ▸) for which a considerable number of complex and versatile networks were described systematically. Open frameworks involved with various cavities such tunnels or cages, especially in phosphates containing mono, di and trivalent cations, are of particular inter­est owing to their potential applications in catalysis (Badrour *et al.*, 2001 ▸) and as immobilizing carriers for various enzymes, *e.g.* CaTi_4_(PO_4_)_6_ (Suzuki *et al.*, 1991 ▸) as well as for their photocatalytic activities in glass-ceramics containing MgTi_4_(PO_4_)_6_ crystals (Fu, 2014 ▸) and ionic-conductivity properties with Ca_l-x_Na_2*x*_Ti_4_(PO_4_)_6_ belonging to the Nasicon structure type (Mentre & Abraham, 1994 ▸). Much attention has been paid to compounds containing six PO_4_ tetra­hedral units with different transition metal/phosphate ratios, *e.g*. Na_4_CaFe_4_(PO_4_)_6_ which adopts the Alluaudite structure in the ideal *C*2/*c* space group (Hidouri *et al.*, 2004 ▸), Ba_3_V4(PO_4_)_6_ which crystallizes as a Langbeinite-type structure (Dross & Glaum, 2004 ▸), CuTi_4_(PO_4_)_6_ which belongs to the Nasicon family (Kasuga *et al.*, 1999 ▸), the silver lead apatite Pb_8_Ag_2_(PO_4_)_6_ (Ternane *et al.*, 2000 ▸), the mixed-valent iron(II/III) phosphate Fe_7_(PO_4_)_6_ (Belik *et al.*, 2000 ▸) and Na_2.5_Y_0.5_Mg_7_(PO_4_)_6_ with a Fillowite-type structure (Jerbi *et al.*, 2010 ▸). Through hydro­thermal processes, and as part of our systematic studies of the crystal alkaline and alkaline earth monophosphates, we have previously succeeded in elaborating a number of compounds with three-dimensional networks featuring distinctive cavities including AgMg_3_(HPO_4_)_2_PO_4_ (Assani *et al.*, 2011 ▸), Sr_2_Mn_3_(HPO_4_)_2_(PO_4_)_2_ (Khmiyas *et al.*, 2013 ▸), SrMn_2_^II^Mn^III^(PO_4_)_3_ (Alhakmi *et al.*, 2013 ▸), NaMg_3_(HPO_4_)_2_PO_4_ (Ould Saleck *et al.*, 2015 ▸). In an extension of our investigations and structural studies of various mono-divalent transition-metal phosphates a new phosphate copper (Cu^II^)-based AgSr_4_Cu_4.5_(PO_4_)_6_ was prepared and successfully characterized. Charge-distribution (CHARDI) (Nespolo *et al.*, 2001 ▸) and bond-valence-sum (BVS) calculations were used for validating the structural model. A careful examination of the literature as well as various databases reveals that the title compound AgSr_4_Cu_4.5_(PO_4_)_6_ is original and furthermore is not related to any family of reported compounds.

## Structural commentary   

**Figure 1:**
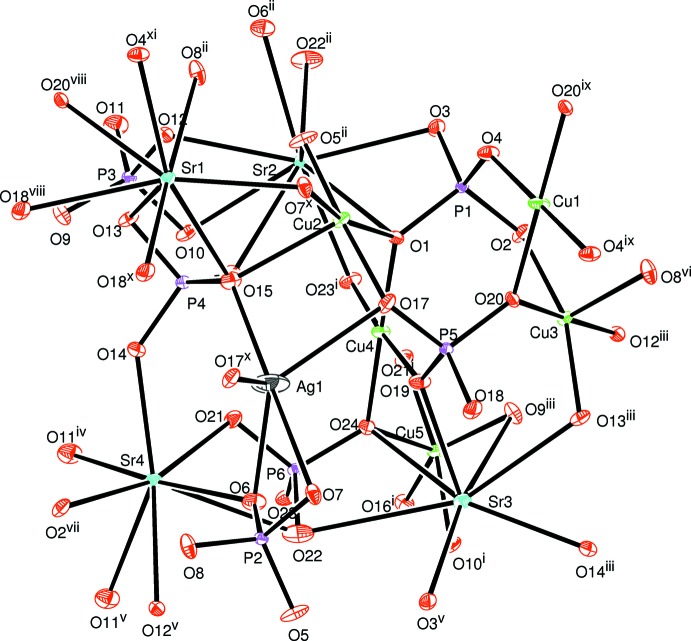
The principal building units in the structure of the title compound. Displacement ellipsoids are drawn at the 50% probability level. Symmetry codes: (i) −*x* + 1, −*y* + 1, −*z*; (ii) −*x*, −*y* + 1, −*z*; (iii) *x*, *y* + 1, *z*; (iv) *x* − 1, *y*, *z*; (v) −*x* + 1, −*y* + 1, −*z* + 1; (vi) *x* + 1, *y*, *z*; (vii) *x*, *y* − 1, *z*; (viii) −*x*, −*y*, −*z* + 1; (ix) −*x*, −*y* + 1, −*z* + 1.

**Figure 2:**
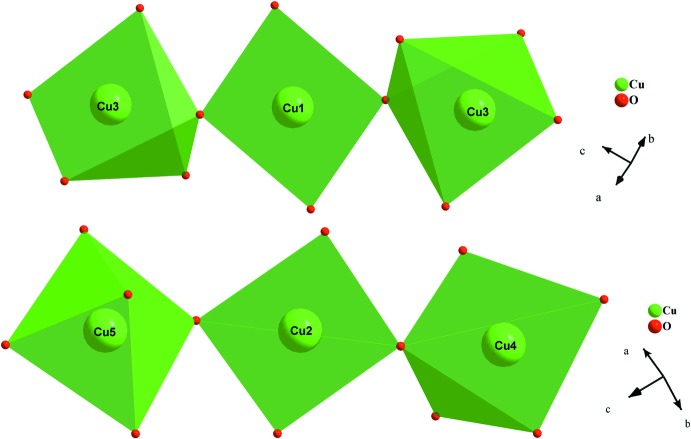
Vertex-sharing [CuO_5_] and CuO_4_ polyhedra forming two [Cu_3_O_12_] trimers.

**Figure 3:**
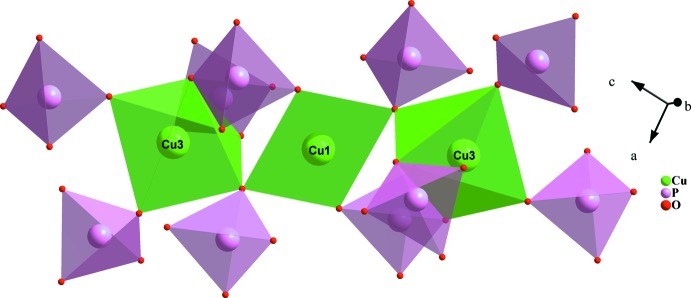
A ribbon, resulting from a Cu3O_5_–Cu1O_4_–Cu3O_5_ trimer connection *via* vertices of PO_4_ tetra­hedra.

**Figure 5:**
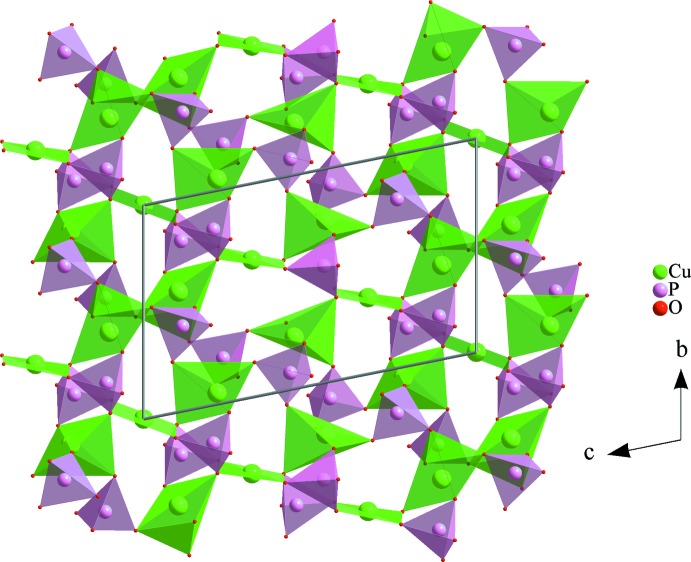
A [Cu_3_O_12_] trimer linked to PO_4_ through corners to form a layer parallel to the (100) plan.

The principal building units of the crystal structure of AgSr_4_Cu_4.5_(PO_4_)_6_ are more or less distorted polyhedra (AgO_5_, CuO_4_, CuO_5_, SrO_8_, SrO_9_) and nearly regular PO_4_ tetra­hedra, as shown in Fig. 1 ▸. In this structure, the copper atoms adopt two different environments: CuO_4_ and CuO_5_. Indeed, Cu1 and Cu2 exhibit a coordination sphere of four oxygen atoms, forming a flattened parallelogram for Cu1O_4_ and a distorted square plane for Cu2O_4_. The other copper atoms Cu3, Cu4 and Cu5 each occupy the centers of CuO_5_ square-based pyramids. A close inspection of the geometrical parameters of Cu3O_5_, Cu4O_5_ and Cu5O_5_ polyhedra reveals that the latter exhibit significant distortion. The phospho­rus atoms are tetra­hedrally coordinated with bond lengths and angles close to those reported for P^5+^ for this geometry. The crystal-structure framework of AgSr_4_Cu_4.5_(PO_4_)_6_ can be viewed as a three-dimensional network of corner-sharing CuO_*n*_ (*n* = 4 or 5) units, thereby forming two types of [Cu_3_O_12_]^18−^ trimers. The first trimer results from the zigzag succession in the following order Cu(4)O_5_ – Cu(2)O_4_ – Cu(5)O_5_. Similarly, the second type of trimer is built up from two-vertex-sharing of a single polyhedra, Cu1O_4_, sandwiched by two neighbouring Cu3O_5_ entities as shown in Fig. 2 ▸. Each oxygen atom of both trimers is connected to a nearly regular PO_4_ tetra­hedron in such a way as to form two different [Cu_3_P_10_O_40_]^24−^ ribbons (see Fig. 3 ▸ and 4 ▸). These adjacent ribbons are linked together through the PO_4_ tetra­hedra, thus building a layer-like [Cu_4.5_(PO_4_)_6_]^9−^ arrangement perpendicular to the [100] direction as shown in Fig. 5 ▸.

**Figure 6:**
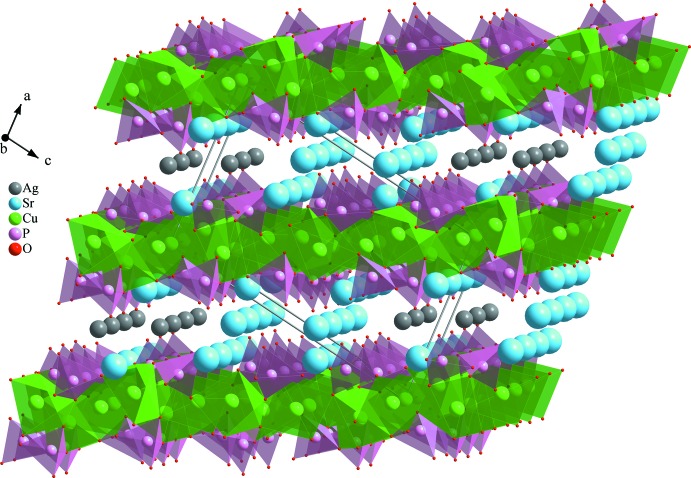
Three dimensional view of AgSr_4_Cu_4.5_(PO_4_)_6_ crystal structure showing Sr^2+^ and Ag^+^ between layers stacked along the [100] direction.

Crystal cohesion and the junction between the stacked layers along the *a*-axis direction are ensured by ionic bonds involving the Sr^2+^ and Ag^+^ cations as shown in Fig. 6 ▸. The insertion of these mono and bivalent cations generates strong inter­actions inducing, consequently, a morphological deformation of the inter­layer space, which explains the manifestation of the distorted sites. This result is confirmed by the CHARDI analysis of the coordination polyhedra by means of the effective coordination number (ECoN; Nespolo, 2016 ▸). The distortion of the metal–oxygen polyhedron becomes stronger when the ECoN value deviates further from the habitual coordination number (CN). This structural particularity is clearly noticeable when examining the numerical values of ECoN and CN for the various SrO_*n*_ (*n* = 8 and 9) and AgO_5_ polyhedra. The differences ECoN (Sr1)/CN(Sr1) = 7.61/8, ECoN (Sr2)/CN(Sr2) = 6.96/8 and ECoN (Sr3)/CN(Sr3) = 6.8/8, reveal an increased distortion in the SrO_8_ groups ranging from the Sr1O_8_ to Sr3O_8_ polyhedra. The Sr2 atom is formally nine-coordinate with bond lengths varying from 2.480 (2) to 2.890 (2) Å. The site hosting Sr4 is very flexible and bulky, resulting in a greatly deformed SrO_9_ polyhedron. The geometry ratio ECoN (Ag1)/CN(Ag1) = 3.93/5 of the Ag1O_5_ polyhedron indicates a distorted square-pyramidal coordination environment. This behaviour can be attributed to the edge or face-sharing between these polyhedral units. This modality of linkage, as well as the ionic radius of Sr^2+^ and Ag^+^, induces a strong cation–cation electrostatic repulsion, which is reflected in the inter­atomic distances and consequently on the repetition of the ionic charge and bond-valence-sum (BVS) values.

The CHARDI analysis method gives the distribution of calculated ECoN numbers of a central cation among all the neighbouring anions (Hoppe, 1979 ▸). The calculation of this number is related directly to the distribution of charges in crystalline structures. The measure of the correctness of the structure (cation ratio) and of the degree of over or under bonding (anion ratio) is performed *via* the evaluation of the inter­nal criterion *q*/*Q* (where *q* is the formal oxidation number and *Q* the computed charge). The charge-distribution method (CD or CHARDI), developed by Hoppe *et al.* (1989 ▸), and the bond –valence (BVS) approach introduced to predict bond lengths in inorganic crystals (Brown, 1977 ▸, 1978 ▸) provide powerful tools for analysis of the connectivity of crystal structures and the validation of structural models. In the present study, both validation tools, BVS and CHARDI, are applied to the structural model of the title compound. Generally, for a well-refined structure, the calculated valences *V*(*i*) obtained by the BVS model and the computed charge *Q*(*i*) according to the CHARDI analysis must be in close agreement with the oxidation number of the atoms. The CHARDI computations were carried out with the *CHARDI2015* program (Nespolo & Guillot, 2016 ▸), while BVS was calculated using *PLATON* (Spek, 2009 ▸). In the asymmetric unit, all atoms are located on general positions (Wyckoff position 2i) of space group *P* 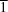 except for Cu1, which is located on a special position (Wyckoff position 1*a*). The distribution of the electric charges at the 40 crystallographic sites of the asymmetric unit shows that the Ag^+^, Sr^2+^, Cu^2+^ and P^5+^ cations fully occupy 16 sites. Otherwise, charge neutrality requires the location of 24 oxygen atoms in the remaining 2i sites. The first results of BVS calculations for Sr3 suggest a valence *V*(Sr3) = 1.900 v.u. for a coordination number CN = 7. This result can be significantly improved by widening the coordination sphere to 3.1410 Å, which allows the integration of a supplemental oxygen, thus inducing valence *V*(Sr3) = 1.962 v.u. The analysis of the data summarized in Table 1 ▸ reveals that the values obtained from charges *Q*(*i*) and bond-valence sums *V*(*i*) of the cations are all compatible with the weighted oxidation number *q*(*i*)·sof(*i*). The minor deviations reported from these parameters with respect to the formal oxidation state are closely related to the distortion level of the occupied sites. Despite these irregularities, all the values of the inter­nal criterion *q*(*i*)/*Q*(*i*) are very close to unity, which confirms the validity of the structural model obtained from the X-ray diffraction data. The convergence of the CHARDI model is evaluated by the mean absolute percentage deviation (MAPD) as shown in the equation below, which measures the agreement between *q*(*i*) and *Q*(*i*) for the whole sets of PC atoms (polyhedron-centring atoms) and of V atoms (the vertex atoms) (Eon & Nespolo, 2015 ▸). For the cationic charges in the structure, we report that the calculated value of MAPD is only 1.7%.
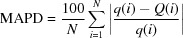
where *N* is the number of polyhedron-centring or vertex atoms in the asymmetric unit.

The calculated anionic charges *Q*(*i*) of oxygen show a lowest deviation of the order of 4.5% with respect to *q*(*i*). These values of MAPD show that the dual description as cation-centred and anion-centred is satisfactory and adequate for the studied structural model. The ratio *q*(*i*)/*Q*(*i*) is approximately equal to 1 in most cases (Table 2 ▸), with some exceptions: *q*(O8)/Q(O8) = 1.16, *q*(O12)/Q(O12) = 0.92 and *q*(O22)/Q(O22) = 1.15. This anomaly of negative-charge repetition could be due to the OUB effect (over–under bonding effect) (Nespolo *et al.*, 1999 ▸), which results from the repulsive inter­actions of the cations located at the centre of the polyhedra. Therefore the anionic charges of oxygen deviate slightly from the ideal value −2. This also explains the variation of cation–anion distances in the various polyhedra in the crystal structure of AgSr_4_Cu_4.5_(PO_4_)_6_.

The plausibility of a crystal-structure model may also be tested by the global instability index (GII) (Salinas-Sanchez *et al.*, 1992 ▸). The calculated value of the GII index measures the deviation of the bond-valence sums from the formal valence *V_i_* averaged over all *N* atoms of the asymmetric unit. For an unstrained structure, GII is below 0.1 v.u. and may approach 0.2 v.u. in a structure with lattice-induced strains (Adams *et al.*, 2004 ▸). Values larger than 0.2 v.u. are typically taken as an indication of the presence of intrinsic strains strong enough to cause instability of the crystal structure (Brown, 1992 ▸). For the crystal structure of the title compound, GII = 0.0944, which indicates high stability and rigidity of the proposed structural model.

## Database survey   

A search in the ICSD database shows that no compounds are currently known in the quaternary system AgO/SrO/CuO/P_2_O_5_. The same is true within the AgO/SrO/P_2_O_5_ ternary system. However, one compound is known in the AgO/CuO/P_2_O_5_ ternary system, *viz.* β-AgCuPO_4_ which crystallizes in the *Pbca* space group (Quarton & Oumba, 1983 ▸). There are seven compounds known in the ternary SrO/CuO/P_2_O_5_ system, *viz.* Sr_9.1_Cu_1.4_(PO_4_)_7_, Sr_3_Cu_3_(PO_4_)_4_ (Belik *et al.*, 2002 ▸; Effenberger, 1999 ▸), Sr_2.88_Cu_3.12_(PO_4_)_4_ (Karanović *et al.*, 2010 ▸), Sr_5_(CuO_2_)_0.333_(PO_4_)_3_ (Kazin *et al.*, 2003 ▸), Sr_2_Cu(PO_4_)_2_, SrCu_2_(PO_4_)_2_ (Belik *et al.*, 2005 ▸) and SrCu(P_2_O_7_) (Moqine *et al.*, 1993 ▸). There is no apparent relation between the structures of these compounds and that of the title compound AgSr_4_Cu_4.5_(PO_4_)_6_.

## Synthesis and crystallization   

Single crystals of the title compound were obtained using the hydro­thermal method with the following mixture of reagents: silver nitrate, strontium nitrate, metallic copper and 85wt% phospho­ric acid in a proportion corresponding to the molar ratio Ag:Cu:Sr:P = 1:3:1:3. The hydro­thermal reaction was conducted in a 23 mL Teflon-lined autoclave with 12 mL of distilled water under autogenous pressure. The vessel was heated to 473 K for 4 d. After being filtered off, washed with distilled water and dried in air, the reaction product consisted of a light-blue crystals in various forms corresponding to the title compound.

## Refinement   

Crystal data, data collection and structure refinement details are summarized in Table 3 ▸. The refinement of the occupation of all atom sites shows full occupancy and leads to the stoichiometric formula AgSr_4_Cu_4.5_(PO_4_)_6_. However, the difference-Fourier map shows two electron-density peaks of intensity 4.05 and −3.87 e Å^−3^ located at 0.63 and 0.59 Å from Ag1, respectively. These rather strong peaks could not be removed using a different integration strategy or another absorption model.

## Supplementary Material

Crystal structure: contains datablock(s) I. DOI: 10.1107/S2056989020000109/vn2155sup1.cif

Structure factors: contains datablock(s) I. DOI: 10.1107/S2056989020000109/vn2155Isup2.hkl

CCDC reference: 1975726

Additional supporting information:  crystallographic information; 3D view; checkCIF report

## Figures and Tables

**Figure 4:**
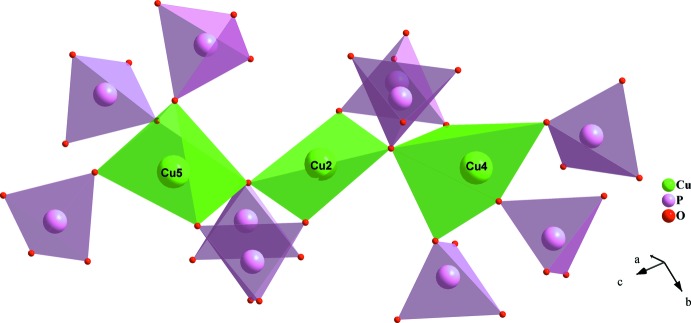
A [Cu_3_O_12_] trimer linked to PO_4_ through corners to build up a ribbon involving Cu4O_5_–Cu2O_4_–Cu5O_5_.

**Table 1:** CHARDI and BVS analysis for the cations in the title compound *q*(*i*) = formal oxidation number; sof(*i*) = site occupancy; CN(*i*) = classical coordination number; *Q*(*i*) = calculated charge; *V*(*i*) = calculated valence; ECoN(*i*) = effective coordination number.

Cation	*q*(*i*)·sof(*i*)	CN(*i*)	ECoN(*i*)	*V*(*i*)	*Q*(*i*)	*q*(*i*)/*Q*(*i*)
Ag1	1	5	3.93	0.998	1.02	0.98
Sr1	2	8	7.61	2.125	2.03	0.98
Sr2	2	8	6.96	2.308	1.99	1.00
Sr3	2	8	6.80	1.962	1.98	1.01
Sr4	2	9	8.07	2.248	2.01	0.99
Cu1	2	4	3.97	1.765	1.92	1.04
Cu2	2	4	3.97	2.000	1.96	1.02
Cu3	2	5	4.55	2.050	1.94	1.03
Cu4	2	5	4.37	2.039	2.00	1.00
Cu5	2	5	4.25	1.957	1.94	1.03
P1	5	4	3.97	4.892	4.91	1.02
P2	5	4	4.00	4.962	5.20	0.96
P3	5	4	3.97	4.974	4.98	1.00
P4	5	4	3.99	4.944	5.04	0.99
P5	5	4	3.97	4.939	4.93	1.01
P6	5	4	3.99	5.016	5.10	0.98

**Table 2:** CHARDI calculation for the oxygen anions in the title compound

Atom	sof(*i*)	*q*(*i*)	*Q*(*i*)	*q*(*i*)/*Q*(*i*)
O1	1	−2	−2.02	0.99
O2	1	−2	−2.07	0.97
O3	1	−2	−1.98	1.01
O4	1	−2	−2.08	0.96
O5	1	−2	−2.12	0.94
O6	1	−2	−1.85	1.08
O7	1	−2	−2.05	0.97
O8	1	−2	−1.73	1.16
O9	1	−2	−1.90	1.05
O10	1	−2	−2.09	0.96
O11	1	−2	−1.92	1.04
O12	1	−2	−2.18	0.92
O13	1	−2	−2.06	0.97
O14	1	−2	−2.00	1.00
O15	1	−2	−1.98	1.01
O16	1	−2	−1.91	1.05
O17	1	−2	−2.05	0.98
O18	1	−2	−1.91	1.05
O19	1	−2	−2.09	0.96
O20	1	−2	−2.09	0.96
O21	1	−2	−2.12	0.94
O22	1	−2	−1.74	1.15
O23	1	−2	−2.05	0.97
O24	1	−2	−2.00	1.00

**Table 3:** Experimental details

Crystal data	
Chemical formula	AgCu_4.50_O_24_P_6_Sr_4_
*M*_r_	1314.08
Crystal system, space group	Triclinic, *P* 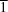
Temperature (K)	296
*a*, *b*, *c* (Å)	9.1070 (1), 9.1514 (1), 13.7259 (2)
α, β, γ (°)	97.498 (1), 98.303 (1), 110.875 (1)
*V* (Å^3^)	1036.97 (2)
*Z*	2
Radiation type	Mo *K*α
μ (mm^−1^)	16.22
Crystal size (mm)	0.30 × 0.27 × 0.21

Data collection	
Diffractometer	Bruker X8 APEXII
Absorption correction	Multi-scan (*SADABS*; Krause *et al.*, 2015 ▸)
*T*_min_, *T*_max_	0.496, 0.747
No. of measured, independent and observed [*I* > 2σ(*I*)] reflections	32671, 8573, 7465
*R*_int_	0.028
(sin θ/λ)_max_ (Å^−1^)	0.806

Refinement	
*R*[*F* ^2^ > 2σ(*F* ^2^)], *wR*(*F* ^2^), *S*	0.028, 0.059, 1.03
No. of reflections	8573
No. of parameters	359
Δρ_max_, Δρ_min_ (e Å^−3^)	4.05, −3.87

Computer programs: *APEX2* and *SAINT* (Bruker, 2009 ▸), *SHELXT2014/7* (Sheldrick, 2015*a* ▸), *SHELXL2014/7* (Sheldrick, 2015*b* ▸), *ORTEP-3 for Windows* (Farrugia, 2012 ▸), *DIAMOND* (Brandenburg, 2006 ▸) and *publCIF* (Westrip, 2010 ▸).

## supplementary crystallographic information

### Crystal data

**Table:**

AgCu_4.50_O_24_P_6_Sr_4_	*Z* = 2
*M**_r_* = 1314.08	*F*(000) = 1223
Triclinic, *P*1	*D*_x_ = 4.209 Mg m^−^^3^
*a* = 9.1070 (1) Å	Mo *K*α radiation, λ = 0.71073 Å
*b* = 9.1514 (1) Å	Cell parameters from 8573 reflections
*c* = 13.7259 (2) Å	θ = 2.4–35.0°
α = 97.498 (1)°	µ = 16.22 mm^−^^1^
β = 98.303 (1)°	*T* = 296 K
γ = 110.875 (1)°	Block, light blue
*V* = 1036.97 (2) Å^3^	0.30 × 0.27 × 0.21 mm

### Data collection

**Table:**

Bruker X8 APEXII diffractometer	8573 independent reflections
Radiation source: fine-focus sealed tube	7465 reflections with *I* > 2σ(*I*)
Graphite monochromator	*R*_int_ = 0.028
φ and ω scans	θ_max_ = 35.0°, θ_min_ = 2.4°
Absorption correction: multi-scan (SADABS; Krause *et al.*, 2015)	*h* = −14→14
*T*_min_ = 0.496, *T*_max_ = 0.747	*k* = −12→14
32671 measured reflections	*l* = −21→20

### Refinement

**Table:**

Refinement on *F*^2^	0 restraints
Least-squares matrix: full	*w* = 1/[σ^2^(*F*_o_^2^) + (0.0206*P*)^2^ + 3.8655*P*] where *P* = (*F*_o_^2^ + 2*F*_c_^2^)/3
*R*[*F*^2^ > 2σ(*F*^2^)] = 0.028	(Δ/σ)_max_ = 0.001
*wR*(*F*^2^) = 0.059	Δρ_max_ = 4.05 e Å^−^^3^
*S* = 1.03	Δρ_min_ = −3.87 e Å^−^^3^
8573 reflections	Extinction correction: SHELXL-2018/3 (Sheldrick, 2015*b*), Fc^*^=kFc[1+0.001xFc^2^λ^3^/sin(2θ)]^-1/4^
359 parameters	Extinction coefficient: 0.00083 (7)

### Special details

**Table:**

Geometry. All esds (except the esd in the dihedral angle between two l.s. planes) are estimated using the full covariance matrix. The cell esds are taken into account individually in the estimation of esds in distances, angles and torsion angles; correlations between esds in cell parameters are only used when they are defined by crystal symmetry. An approximate (isotropic) treatment of cell esds is used for estimating esds involving l.s. planes.

### Fractional atomic coordinates and isotropic or equivalent isotropic displacement parameters (Å^2^)

**Table:**

	*x*	*y*	*z*	*U*_iso_*/*U*_eq_	
Ag1	0.60647 (3)	0.58776 (4)	0.10399 (2)	0.02934 (7)	
Sr1	0.28824 (3)	0.81800 (3)	0.01654 (2)	0.00676 (5)	
Sr2	0.07030 (3)	0.54033 (3)	0.33636 (2)	0.00652 (5)	
Sr3	0.63381 (3)	0.17215 (3)	0.32562 (2)	0.00823 (5)	
Sr4	0.87164 (3)	0.85230 (3)	0.35406 (2)	0.00832 (5)	
Cu1	0.000000	0.000000	0.000000	0.00876 (9)	
Cu2	0.19756 (4)	0.43833 (4)	0.10393 (2)	0.00679 (6)	
Cu3	0.17555 (4)	−0.05281 (4)	0.22039 (2)	0.00602 (6)	
Cu4	0.35439 (4)	0.35727 (4)	0.32546 (2)	0.00693 (6)	
Cu5	0.51284 (4)	0.24192 (4)	0.54770 (2)	0.00725 (6)	
P1	−0.00318 (8)	0.17911 (8)	0.20922 (5)	0.00451 (11)	
P2	0.93662 (8)	0.60245 (8)	0.11620 (5)	0.00507 (11)	
P3	0.24288 (8)	0.92189 (8)	0.44052 (5)	0.00544 (11)	
P4	0.44974 (8)	0.78964 (8)	0.23844 (5)	0.00542 (11)	
P5	0.37042 (8)	0.20301 (8)	0.11200 (5)	0.00480 (11)	
P6	0.69847 (8)	0.54683 (8)	0.45908 (5)	0.00475 (11)	
O1	0.1628 (2)	0.3232 (2)	0.22180 (14)	0.0064 (3)	
O2	0.0314 (2)	0.0493 (2)	0.25727 (15)	0.0091 (3)	
O3	−0.1119 (2)	0.2403 (2)	0.26131 (15)	0.0093 (3)	
O4	−0.0762 (2)	0.1176 (2)	0.09618 (14)	0.0092 (3)	
O5	1.0559 (3)	0.5276 (3)	0.15350 (15)	0.0128 (4)	
O6	0.8509 (3)	0.6235 (3)	0.20225 (15)	0.0112 (4)	
O7	0.8129 (2)	0.4854 (2)	0.02384 (14)	0.0092 (4)	
O8	1.0258 (3)	0.7603 (2)	0.08664 (15)	0.0116 (4)	
O9	0.3837 (2)	1.0680 (2)	0.43059 (15)	0.0106 (4)	
O10	0.2988 (2)	0.7963 (2)	0.48272 (14)	0.0089 (3)	
O11	0.1335 (3)	0.9560 (3)	0.50496 (16)	0.0129 (4)	
O12	0.1398 (2)	0.8387 (2)	0.33335 (13)	0.0065 (3)	
O13	0.3714 (2)	0.9110 (2)	0.21547 (14)	0.0087 (3)	
O14	0.6258 (2)	0.8917 (2)	0.28321 (16)	0.0107 (4)	
O15	0.4209 (3)	0.6805 (2)	0.13724 (14)	0.0099 (4)	
O16	0.3742 (2)	0.6848 (2)	0.31152 (14)	0.0089 (3)	
O17	0.3381 (3)	0.3334 (2)	0.06366 (15)	0.0094 (4)	
O18	0.4811 (3)	0.1406 (2)	0.06409 (16)	0.0108 (4)	
O19	0.4412 (2)	0.2650 (2)	0.22584 (14)	0.0092 (4)	
O20	0.2067 (2)	0.0587 (2)	0.09995 (14)	0.0070 (3)	
O21	0.6559 (2)	0.6837 (2)	0.42809 (15)	0.0094 (3)	
O22	0.8374 (3)	0.5349 (3)	0.41485 (16)	0.0160 (4)	
O23	0.7430 (2)	0.5693 (2)	0.57441 (14)	0.0090 (3)	
O24	0.5494 (2)	0.3884 (2)	0.42094 (14)	0.0083 (3)	

### Atomic displacement parameters (Å^2^)

**Table:**

	*U*^11^	*U*^22^	*U*^33^	*U*^12^	*U*^13^	*U*^23^
Ag1	0.01409 (12)	0.04266 (17)	0.03685 (16)	0.01756 (12)	0.00787 (11)	0.00447 (13)
Sr1	0.00637 (10)	0.00740 (10)	0.00765 (10)	0.00327 (8)	0.00283 (8)	0.00199 (8)
Sr2	0.00621 (10)	0.00615 (10)	0.00775 (10)	0.00271 (8)	0.00232 (8)	0.00124 (7)
Sr3	0.00784 (11)	0.00971 (10)	0.00884 (10)	0.00481 (9)	0.00298 (8)	0.00198 (8)
Sr4	0.00637 (11)	0.01015 (11)	0.01038 (10)	0.00413 (9)	0.00398 (8)	0.00341 (8)
Cu1	0.0054 (2)	0.0135 (2)	0.00640 (18)	0.00484 (17)	−0.00058 (15)	−0.00297 (16)
Cu2	0.00818 (15)	0.00827 (14)	0.00676 (13)	0.00519 (12)	0.00333 (11)	0.00329 (10)
Cu3	0.00630 (14)	0.00746 (13)	0.00649 (13)	0.00409 (11)	0.00282 (11)	0.00295 (10)
Cu4	0.00585 (14)	0.00973 (14)	0.00516 (12)	0.00403 (11)	0.00015 (10)	−0.00060 (10)
Cu5	0.00636 (14)	0.00897 (14)	0.00652 (13)	0.00391 (11)	0.00033 (11)	0.00019 (10)
P1	0.0042 (3)	0.0048 (3)	0.0052 (2)	0.0022 (2)	0.0016 (2)	0.0010 (2)
P2	0.0043 (3)	0.0056 (3)	0.0050 (2)	0.0017 (2)	0.0006 (2)	0.0011 (2)
P3	0.0046 (3)	0.0056 (3)	0.0058 (3)	0.0020 (2)	0.0006 (2)	0.0001 (2)
P4	0.0047 (3)	0.0063 (3)	0.0053 (3)	0.0024 (2)	0.0007 (2)	0.0012 (2)
P5	0.0042 (3)	0.0051 (3)	0.0054 (2)	0.0018 (2)	0.0018 (2)	0.0010 (2)
P6	0.0043 (3)	0.0060 (3)	0.0048 (2)	0.0024 (2)	0.0017 (2)	0.0016 (2)
O1	0.0043 (8)	0.0073 (8)	0.0071 (8)	0.0017 (6)	0.0007 (6)	0.0020 (6)
O2	0.0109 (9)	0.0090 (8)	0.0119 (8)	0.0065 (7)	0.0058 (7)	0.0056 (7)
O3	0.0081 (9)	0.0080 (8)	0.0137 (9)	0.0042 (7)	0.0056 (7)	0.0016 (7)
O4	0.0083 (9)	0.0128 (9)	0.0067 (8)	0.0059 (7)	−0.0001 (7)	−0.0005 (7)
O5	0.0156 (10)	0.0221 (11)	0.0095 (8)	0.0159 (9)	0.0041 (7)	0.0058 (8)
O6	0.0088 (9)	0.0165 (10)	0.0098 (8)	0.0063 (8)	0.0038 (7)	0.0011 (7)
O7	0.0097 (9)	0.0065 (8)	0.0071 (8)	−0.0004 (7)	−0.0015 (7)	0.0002 (6)
O8	0.0122 (10)	0.0079 (8)	0.0110 (9)	−0.0011 (7)	0.0034 (7)	0.0023 (7)
O9	0.0091 (9)	0.0083 (8)	0.0096 (8)	−0.0017 (7)	0.0013 (7)	0.0006 (7)
O10	0.0104 (9)	0.0114 (9)	0.0080 (8)	0.0072 (7)	0.0019 (7)	0.0038 (7)
O11	0.0112 (10)	0.0164 (10)	0.0120 (9)	0.0067 (8)	0.0040 (7)	−0.0004 (7)
O12	0.0061 (8)	0.0075 (8)	0.0052 (7)	0.0020 (7)	0.0002 (6)	0.0018 (6)
O13	0.0092 (9)	0.0110 (9)	0.0095 (8)	0.0069 (7)	0.0027 (7)	0.0043 (7)
O14	0.0063 (9)	0.0086 (8)	0.0157 (9)	0.0020 (7)	−0.0011 (7)	0.0032 (7)
O15	0.0102 (9)	0.0124 (9)	0.0073 (8)	0.0061 (7)	0.0005 (7)	−0.0014 (7)
O16	0.0084 (9)	0.0106 (8)	0.0069 (8)	0.0023 (7)	0.0011 (7)	0.0035 (6)
O17	0.0112 (9)	0.0090 (8)	0.0125 (9)	0.0063 (7)	0.0067 (7)	0.0055 (7)
O18	0.0102 (9)	0.0108 (9)	0.0152 (9)	0.0063 (7)	0.0077 (7)	0.0028 (7)
O19	0.0079 (9)	0.0128 (9)	0.0063 (8)	0.0051 (7)	−0.0007 (7)	−0.0018 (7)
O20	0.0049 (8)	0.0061 (8)	0.0081 (8)	0.0004 (6)	0.0001 (6)	0.0017 (6)
O21	0.0094 (9)	0.0088 (8)	0.0132 (9)	0.0048 (7)	0.0048 (7)	0.0063 (7)
O22	0.0108 (10)	0.0293 (12)	0.0141 (9)	0.0117 (9)	0.0087 (8)	0.0067 (9)
O23	0.0108 (9)	0.0137 (9)	0.0048 (7)	0.0076 (7)	0.0007 (7)	0.0015 (6)
O24	0.0068 (8)	0.0070 (8)	0.0095 (8)	0.0020 (7)	−0.0012 (7)	0.0007 (6)

### Geometric parameters (Å, º)

**Table:**

Ag1—O15	2.220 (2)	Cu2—O17	1.951 (2)
Ag1—O6	2.319 (2)	Cu2—O7^i^	1.9723 (19)
Ag1—O17^i^	2.565 (2)	Cu2—O1	2.0450 (19)
Ag1—O17	2.630 (2)	Cu2—O15	2.348 (2)
Ag1—O7	2.684 (2)	Cu3—O13^vii^	1.936 (2)
Sr1—O18^i^	2.449 (2)	Cu3—O2	1.948 (2)
Sr1—O7^i^	2.5484 (19)	Cu3—O12^vii^	1.9528 (18)
Sr1—O4^ii^	2.580 (2)	Cu3—O20	2.0551 (19)
Sr1—O8^iii^	2.610 (2)	Cu3—O8^xi^	2.225 (2)
Sr1—O15	2.615 (2)	Cu4—O23^v^	1.914 (2)
Sr1—O13	2.6628 (19)	Cu4—O19	1.922 (2)
Sr1—O20^iv^	2.7325 (19)	Cu4—O24	1.9553 (19)
Sr1—O18^iv^	2.774 (2)	Cu4—O1	1.9866 (19)
Sr2—O5^iii^	2.480 (2)	Cu5—O21^v^	1.942 (2)
Sr2—O22^iii^	2.502 (2)	Cu5—O10^v^	1.959 (2)
Sr2—O23^v^	2.517 (2)	Cu5—O16^v^	1.9591 (19)
Sr2—O12	2.5813 (19)	Cu5—O9^vii^	1.988 (2)
Sr2—O3	2.622 (2)	Cu5—O24	2.322 (2)
Sr2—O16	2.703 (2)	P1—O3	1.516 (2)
Sr2—O1	2.8087 (19)	P1—O2	1.535 (2)
Sr2—O10	2.819 (2)	P1—O4	1.541 (2)
Sr2—O6^iii^	2.890 (2)	P1—O1	1.581 (2)
Sr3—O3^vi^	2.498 (2)	P2—O8	1.524 (2)
Sr3—O19	2.520 (2)	P2—O6	1.535 (2)
Sr3—O14^vii^	2.529 (2)	P2—O5	1.540 (2)
Sr3—O10^v^	2.5672 (19)	P2—O7	1.548 (2)
Sr3—O24	2.630 (2)	P3—O11	1.508 (2)
Sr3—O13^vii^	2.766 (2)	P3—O9	1.525 (2)
Sr3—O9^vii^	2.816 (2)	P3—O10	1.555 (2)
Sr3—O22	3.139 (3)	P3—O12	1.5572 (19)
Sr3—O11^v^	3.511 (2)	P4—O14	1.523 (2)
Sr4—O11^viii^	2.455 (2)	P4—O15	1.530 (2)
Sr4—O21	2.470 (2)	P4—O16	1.543 (2)
Sr4—O14	2.475 (2)	P4—O13	1.560 (2)
Sr4—O2^ix^	2.539 (2)	P5—O18	1.509 (2)
Sr4—O12^vi^	2.543 (2)	P5—O17	1.533 (2)
Sr4—O6	2.688 (2)	P5—O19	1.548 (2)
Sr4—O11^vi^	2.707 (2)	P5—O20	1.570 (2)
Sr4—O22	3.048 (2)	P5—O1	2.988 (2)
Cu1—O4^x^	1.9515 (19)	P6—O22	1.512 (2)
Cu1—O4	1.9515 (19)	P6—O21	1.529 (2)
Cu1—O20^x^	2.0138 (19)	P6—O23	1.544 (2)
Cu1—O20	2.0138 (19)	P6—O24	1.553 (2)
Cu2—O5^iii^	1.912 (2)		
			
O15—Ag1—O6	130.30 (7)	O6—Sr4—O22	65.95 (6)
O15—Ag1—O17^i^	104.15 (7)	O11^vi^—Sr4—O22	80.99 (6)
O6—Ag1—O17^i^	107.23 (7)	O4^x^—Cu1—O4	180.0
O15—Ag1—O17	75.47 (7)	O4^x^—Cu1—O20^x^	90.19 (8)
O6—Ag1—O17	127.71 (7)	O4—Cu1—O20^x^	89.82 (8)
O17^i^—Ag1—O17	107.34 (5)	O4^x^—Cu1—O20	89.82 (8)
O15—Ag1—O7	167.66 (7)	O4—Cu1—O20	90.18 (8)
O6—Ag1—O7	59.96 (6)	O20^x^—Cu1—O20	180.0
O17^i^—Ag1—O7	64.01 (6)	O4^x^—Cu1—O8^i^	104.34 (7)
O17—Ag1—O7	103.95 (6)	O4—Cu1—O8^i^	75.66 (7)
O18^i^—Sr1—O7^i^	94.81 (7)	O20^x^—Cu1—O8^i^	65.34 (7)
O18^i^—Sr1—O4^ii^	108.26 (7)	O20—Cu1—O8^i^	114.66 (7)
O7^i^—Sr1—O4^ii^	104.05 (6)	O4^x^—Cu1—O8^xi^	75.66 (7)
O18^i^—Sr1—O8^iii^	174.83 (7)	O4—Cu1—O8^xi^	104.34 (7)
O7^i^—Sr1—O8^iii^	82.72 (7)	O20^x^—Cu1—O8^xi^	114.66 (7)
O4^ii^—Sr1—O8^iii^	68.14 (7)	O20—Cu1—O8^xi^	65.34 (7)
O18^i^—Sr1—O15	86.11 (7)	O8^i^—Cu1—O8^xi^	180.00 (8)
O7^i^—Sr1—O15	62.56 (6)	O5^iii^—Cu2—O17	174.10 (9)
O4^ii^—Sr1—O15	161.76 (7)	O5^iii^—Cu2—O7^i^	95.24 (9)
O8^iii^—Sr1—O15	96.71 (7)	O17—Cu2—O7^i^	90.39 (8)
O18^i^—Sr1—O13	113.18 (7)	O5^iii^—Cu2—O1	82.50 (8)
O7^i^—Sr1—O13	107.77 (6)	O17—Cu2—O1	91.63 (8)
O4^ii^—Sr1—O13	124.26 (6)	O7^i^—Cu2—O1	168.00 (8)
O8^iii^—Sr1—O13	71.95 (6)	O5^iii^—Cu2—O15	95.57 (9)
O15—Sr1—O13	55.45 (6)	O17—Cu2—O15	87.46 (8)
O18^i^—Sr1—O20^iv^	124.05 (6)	O7^i^—Cu2—O15	76.16 (7)
O7^i^—Sr1—O20^iv^	141.00 (6)	O1—Cu2—O15	115.74 (7)
O4^ii^—Sr1—O20^iv^	63.53 (6)	O5^iii^—Cu2—O8^i^	97.38 (8)
O8^iii^—Sr1—O20^iv^	58.28 (6)	O17—Cu2—O8^i^	84.24 (8)
O15—Sr1—O20^iv^	118.08 (6)	O7^i^—Cu2—O8^i^	56.67 (7)
O13—Sr1—O20^iv^	62.82 (6)	O1—Cu2—O8^i^	111.79 (7)
O18^i^—Sr1—O18^iv^	71.86 (7)	O15—Cu2—O8^i^	131.91 (6)
O7^i^—Sr1—O18^iv^	163.91 (6)	O5^iii^—Cu2—O4	83.95 (8)
O4^ii^—Sr1—O18^iv^	89.03 (6)	O17—Cu2—O4	92.22 (7)
O8^iii^—Sr1—O18^iv^	111.27 (6)	O7^i^—Cu2—O4	113.28 (7)
O15—Sr1—O18^iv^	106.49 (6)	O1—Cu2—O4	54.83 (6)
O13—Sr1—O18^iv^	70.98 (6)	O15—Cu2—O4	170.55 (6)
O20^iv^—Sr1—O18^iv^	53.59 (6)	O8^i^—Cu2—O4	57.36 (5)
O5^iii^—Sr2—O22^iii^	121.69 (7)	O13^vii^—Cu3—O2	160.18 (9)
O5^iii^—Sr2—O23^v^	116.94 (6)	O13^vii^—Cu3—O12^vii^	91.65 (8)
O22^iii^—Sr2—O23^v^	115.45 (7)	O2—Cu3—O12^vii^	88.14 (8)
O5^iii^—Sr2—O12	81.20 (7)	O13^vii^—Cu3—O20	89.56 (8)
O22^iii^—Sr2—O12	89.49 (7)	O2—Cu3—O20	91.05 (8)
O23^v^—Sr2—O12	124.57 (6)	O12^vii^—Cu3—O20	178.46 (8)
O5^iii^—Sr2—O3	77.65 (7)	O13^vii^—Cu3—O8^xi^	96.00 (8)
O22^iii^—Sr2—O3	83.32 (7)	O2—Cu3—O8^xi^	103.27 (9)
O23^v^—Sr2—O3	84.85 (7)	O12^vii^—Cu3—O8^xi^	104.10 (8)
O12—Sr2—O3	149.55 (6)	O20—Cu3—O8^xi^	74.82 (7)
O5^iii^—Sr2—O16	73.18 (7)	O13^vii^—Cu3—O19	71.97 (7)
O22^iii^—Sr2—O16	152.12 (7)	O2—Cu3—O19	92.23 (7)
O23^v^—Sr2—O16	68.53 (6)	O12^vii^—Cu3—O19	126.04 (7)
O12—Sr2—O16	68.48 (6)	O20—Cu3—O19	55.29 (6)
O3—Sr2—O16	124.31 (6)	O8^xi^—Cu3—O19	127.99 (6)
O5^iii^—Sr2—O1	58.77 (6)	O13^vii^—Cu3—O9^vii^	72.67 (7)
O22^iii^—Sr2—O1	137.87 (7)	O2—Cu3—O9^vii^	91.22 (7)
O23^v^—Sr2—O1	61.68 (6)	O12^vii^—Cu3—O9^vii^	54.82 (7)
O12—Sr2—O1	128.37 (6)	O20—Cu3—O9^vii^	126.52 (7)
O3—Sr2—O1	54.80 (6)	O8^xi^—Cu3—O9^vii^	154.46 (7)
O16—Sr2—O1	69.52 (6)	O19—Cu3—O9^vii^	71.23 (5)
O5^iii^—Sr2—O10	122.96 (7)	O23^v^—Cu4—O19	175.03 (9)
O22^iii^—Sr2—O10	94.73 (7)	O23^v^—Cu4—O24	94.12 (8)
O23^v^—Sr2—O10	73.63 (6)	O19—Cu4—O24	86.62 (8)
O12—Sr2—O10	54.70 (6)	O23^v^—Cu4—O1	89.30 (8)
O3—Sr2—O10	155.20 (6)	O19—Cu4—O1	90.03 (8)
O16—Sr2—O10	58.88 (6)	O24—Cu4—O1	176.54 (8)
O1—Sr2—O10	120.87 (6)	O23^v^—Cu4—O16	70.33 (7)
O5^iii^—Sr2—O6^iii^	54.03 (6)	O19—Cu4—O16	114.25 (7)
O22^iii^—Sr2—O6^iii^	70.73 (6)	O24—Cu4—O16	104.78 (7)
O23^v^—Sr2—O6^iii^	169.49 (6)	O1—Cu4—O16	75.83 (7)
O12—Sr2—O6^iii^	62.19 (6)	O23^v^—Cu4—O2	87.24 (7)
O3—Sr2—O6^iii^	87.58 (6)	O19—Cu4—O2	88.45 (7)
O16—Sr2—O6^iii^	110.36 (6)	O24—Cu4—O2	128.42 (7)
O1—Sr2—O6^iii^	107.91 (6)	O1—Cu4—O2	52.25 (6)
O10—Sr2—O6^iii^	115.23 (6)	O16—Cu4—O2	123.74 (5)
O3^vi^—Sr3—O19	110.38 (7)	O21^v^—Cu5—O10^v^	169.90 (9)
O3^vi^—Sr3—O14^vii^	82.58 (7)	O21^v^—Cu5—O16^v^	92.79 (9)
O19—Sr3—O14^vii^	122.19 (6)	O10^v^—Cu5—O16^v^	87.76 (8)
O3^vi^—Sr3—O10^v^	108.90 (7)	O21^v^—Cu5—O9^vii^	97.24 (9)
O19—Sr3—O10^v^	127.01 (6)	O10^v^—Cu5—O9^vii^	86.98 (9)
O14^vii^—Sr3—O10^v^	96.76 (6)	O16^v^—Cu5—O9^vii^	151.10 (9)
O3^vi^—Sr3—O24	122.99 (6)	O21^v^—Cu5—O24	87.84 (8)
O19—Sr3—O24	62.15 (6)	O10^v^—Cu5—O24	83.75 (8)
O14^vii^—Sr3—O24	152.48 (7)	O16^v^—Cu5—O24	126.58 (8)
O10^v^—Sr3—O24	67.04 (6)	O9^vii^—Cu5—O24	80.99 (8)
O3^vi^—Sr3—O13^vii^	117.02 (6)	O21^v^—Cu5—O23	91.10 (7)
O19—Sr3—O13^vii^	70.32 (6)	O10^v^—Cu5—O23	79.53 (7)
O14^vii^—Sr3—O13^vii^	54.77 (6)	O16^v^—Cu5—O23	71.14 (7)
O10^v^—Sr3—O13^vii^	119.03 (6)	O9^vii^—Cu5—O23	135.31 (7)
O24—Sr3—O13^vii^	112.29 (6)	O24—Cu5—O23	55.44 (6)
O3^vi^—Sr3—O9^vii^	166.81 (6)	O21^v^—Cu5—O14^v^	70.52 (7)
O19—Sr3—O9^vii^	82.79 (6)	O10^v^—Cu5—O14^v^	117.73 (7)
O14^vii^—Sr3—O9^vii^	90.80 (6)	O16^v^—Cu5—O14^v^	56.61 (7)
O10^v^—Sr3—O9^vii^	60.41 (6)	O9^vii^—Cu5—O14^v^	101.64 (7)
O24—Sr3—O9^vii^	62.03 (6)	O24—Cu5—O14^v^	158.36 (6)
O13^vii^—Sr3—O9^vii^	66.93 (6)	O23—Cu5—O14^v^	122.40 (5)
O3^vi^—Sr3—O22	73.38 (6)	O3—P1—O2	111.77 (11)
O19—Sr3—O22	86.10 (6)	O3—P1—O4	110.53 (12)
O14^vii^—Sr3—O22	148.30 (6)	O2—P1—O4	110.81 (11)
O10^v^—Sr3—O22	72.66 (6)	O3—P1—O1	107.91 (11)
O24—Sr3—O22	50.51 (6)	O2—P1—O1	107.36 (11)
O13^vii^—Sr3—O22	156.23 (6)	O4—P1—O1	108.30 (11)
O9^vii^—Sr3—O22	108.16 (6)	O8—P2—O6	112.23 (12)
O3^vi^—Sr3—O11^v^	78.24 (6)	O8—P2—O5	110.05 (13)
O19—Sr3—O11^v^	170.75 (6)	O6—P2—O5	106.61 (12)
O14^vii^—Sr3—O11^v^	61.04 (6)	O8—P2—O7	109.64 (11)
O10^v^—Sr3—O11^v^	44.55 (6)	O6—P2—O7	109.63 (12)
O24—Sr3—O11^v^	110.69 (5)	O5—P2—O7	108.57 (12)
O13^vii^—Sr3—O11^v^	109.31 (5)	O11—P3—O9	115.26 (12)
O9^vii^—Sr3—O11^v^	88.58 (6)	O11—P3—O10	107.09 (12)
O22—Sr3—O11^v^	93.45 (6)	O9—P3—O10	112.23 (12)
O11^viii^—Sr4—O21	77.94 (7)	O11—P3—O12	107.68 (12)
O11^viii^—Sr4—O14	80.40 (7)	O9—P3—O12	107.88 (11)
O21—Sr4—O14	74.02 (7)	O10—P3—O12	106.25 (11)
O11^viii^—Sr4—O2^ix^	98.52 (7)	O14—P4—O15	114.18 (12)
O21—Sr4—O2^ix^	163.95 (7)	O14—P4—O16	110.49 (11)
O14—Sr4—O2^ix^	89.98 (7)	O15—P4—O16	108.08 (12)
O11^viii^—Sr4—O12^vi^	118.73 (7)	O14—P4—O13	104.92 (11)
O21—Sr4—O12^vi^	130.97 (6)	O15—P4—O13	105.27 (11)
O14—Sr4—O12^vi^	149.08 (7)	O16—P4—O13	113.93 (11)
O2^ix^—Sr4—O12^vi^	64.54 (6)	O18—P5—O17	113.12 (11)
O11^viii^—Sr4—O6	174.63 (7)	O18—P5—O19	109.40 (12)
O21—Sr4—O6	96.83 (7)	O17—P5—O19	110.47 (11)
O14—Sr4—O6	97.07 (7)	O18—P5—O20	107.49 (11)
O2^ix^—Sr4—O6	86.17 (6)	O17—P5—O20	108.67 (11)
O12^vi^—Sr4—O6	65.64 (6)	O19—P5—O20	107.49 (11)
O11^viii^—Sr4—O11^vi^	65.85 (8)	O18—P5—O1	175.64 (9)
O21—Sr4—O11^vi^	103.32 (7)	O17—P5—O1	70.46 (8)
O14—Sr4—O11^vi^	145.70 (7)	O19—P5—O1	66.55 (8)
O2^ix^—Sr4—O11^vi^	89.13 (7)	O20—P5—O1	72.96 (8)
O12^vi^—Sr4—O11^vi^	56.15 (6)	O22—P6—O21	110.56 (12)
O6—Sr4—O11^vi^	117.07 (6)	O22—P6—O23	109.07 (12)
O11^viii^—Sr4—O22	111.04 (7)	O21—P6—O23	111.36 (11)
O21—Sr4—O22	52.59 (6)	O22—P6—O24	109.57 (13)
O14—Sr4—O22	118.68 (6)	O21—P6—O24	108.90 (11)
O2^ix^—Sr4—O22	141.14 (6)	O23—P6—O24	107.32 (11)
O12^vi^—Sr4—O22	79.02 (6)		

Symmetry codes: (i) −*x*+1, −*y*+1, −*z*; (ii) −*x*, −*y*+1, −*z*; (iii) *x*−1, *y*, *z*; (iv) *x*, *y*+1, *z*; (v) −*x*+1, −*y*+1, −*z*+1; (vi) *x*+1, *y*, *z*; (vii) *x*, *y*−1, *z*; (viii) −*x*+1, −*y*+2, −*z*+1; (ix) *x*+1, *y*+1, *z*; (x) −*x*, −*y*, −*z*; (xi) *x*−1, *y*−1, *z*.
